# Buffalo Colleges

**Published:** 1885-10

**Authors:** 


### BUFFALO COLLEGES.

Before this shall have reached our readers, the two Buffalo Medical Colleges will be in full operation.

The Buffalo Medical College having opened its fall term last week, the course opens with a fair attendance. Dr. Pohlman made the opening address, which was well received by those present. The faculty is the same as before, with the exception of the professor of physiology, which change was announced in a recent number of the JOURNAL.

The Niagara Medical College opened its doors on Wednesday evening. As our readers know, this college begins its third annual course of lectures, but the first in the new building. It was taken possession of last January. Prof. Thomas Lothrop, Vice-President of the Faculty, gave the opening address. It was well suited to the occasion, and brought forth many rounds of applause from the goodly crowd, composed of doctors, students and other friends, which had come together on this auspicious occasion.

The building was open for inspection, and to say that it is perfect in all its appointments, is but feeble praise. We noticed first the spacious, well-lighted and well-ventilated amphitheater. The large and comfortably-arranged lecture-room, the complete chemical laboratory, the convenient physiological laboratory, where each student has a space at the tables large enough to place his instruments and to manipulate his microscope. Just across the hall is the dissecting-room, light, airy and well adapted to this use; the ventilation so perfect, that it is said there is *never* that unpleasant odor so often noticed in these rooms. There is a gaslight over each table; there is plenty of hot and cold water, and a retiring-room. There is no dissecting-room in this country more complete. What more could be added? No wonder it excites our admiration. Near this is the prosector's room; in one corner of it is a lift, by which subjects can b lowered to the lecture-room below.

We noticed, too, that the *comfort* of the students is cared for. There is a reading or assembly room; cheerful and comfortable, with lockers along the wall, one of which is given to each student to use during the course. From this room is the staircase leading to the wash-rooms and closets below, which are perfect in every detail. The building is heated by steam, and well ventilated. The location is all that could be desired, being one block from the Grosvenor library. The latter contains a good medical collection, which is free, and any student may go and spend in study the hours not otherwise occupied, and yet be but one minute's walk from the college. Just opposite, is the Young Men's Association library—now being erected—which has also a medical department, and can be utilized for a nominal sum.

We learned from the Secretary of the college that there is every prospect of a large class for this term. Most of the new matriculants are college or high-school graduates, and all have a good English education. "This can be seen at once by a glance at the class," said a visitor. Many students have decided to take a four years' course, while all are required to take three. We are surprised at the rapid advance of this college, especially when we consider that all the forces of a certain set of men have been at work to break it down in its very beginning, but, as we anticipated, these very attacks have been the means of making the college many warm friends, and it is, in this short space of time, a grand success. It proves that the profession is in favor of higher medical education, and that they will sustain and assist this college as long as they have so noble an aim. It proves that not the profession alone, but the people in this community, will cherish it. Doctors, students, laymen, all recognize the fact that thorough medical education is necessary for the success of the doctor and for the well-being of the community.

